# Exposure to climate-related stressors undermines mental health in the Kurdistan Region of Iraq: a cross-sectional study

**DOI:** 10.3389/fpubh.2025.1719584

**Published:** 2026-01-12

**Authors:** Jan Ilhan Kizilhan, Sumaia Al-Ghurbani, Jonathan Uricher, Zelal Ag, Ibraheem Khalil Musa, Bohar Suleiman Isa

**Affiliations:** 1 Institute for Transcultural Health Science, Baden-Wuerttemberg Cooperative State University (DHBW), Stuttgart, Germany; 2 Institute for Psychotherapy and Psychotraumatology, University of Dohuk, Duhok, Iraq

**Keywords:** climate change, developing countries, extreme weather events, Iraq, Kurdistan region of Iraq, mental health, Middle East, vulnerable populations

## Abstract

**Introduction:**

Climate-related events such as droughts, extreme heat, and flooding intensify pressures on psychological well-being, particularly in vulnerable, low-resource, and conflict-affected settings. Iraq ranks as the world’s fifth most climate-vulnerable country, and the Kurdistan Region has been increasingly affected by frequent and intense extreme weather events that threaten livelihoods and community stability. Despite rising exposure, the mental health impacts of climate change in the Kurdistan Region remain largely understudied. This study investigates the effects of climate change–related stressors and extreme weather events on mental health through robust analytical methods, yielding the first rigorous empirical evidence from the region.

**Methods:**

An analytical cross-sectional study was conducted with a sample of 618 participants aged ≥18 years, residing in urban and rural areas of three governorates in the Kurdistan Region of Iraq: Duhok, Erbil, and Sulaymaniyah. Data were collected using a questionnaire that integrated measures from several validated psychological scales, including the PHQ-8, GAD-7, K10, PCL-5, and CC-MMDS. The data were analyzed using independent t-tests and analysis of variance (ANOVA) for group comparisons, and multiple linear regression for associations.

**Results:**

The final sample included N = 608 adults aged 18–75 years (51.6% male, 48.4% female), recruited from Duhok (32.9%), Erbil (33.9%), and Sulaymaniyah (33.2%). Descriptive analysis identified heatwaves, droughts, and dust storms as the most frequently reported extreme weather events. Multiple linear regression analyses revealed that exposure to extreme weather events significantly predicted increased posttraumatic stress disorder symptom levels (*β* = 0.23, *p* = 0.008). Climate-related homelessness was also significantly associated with increased symptoms of depression (*β* = 0.38, *p* = 0.014), anxiety (*β* = 0.42, *p* = 0.007), psychological distress (*β* = 0.32, *p* = 0.042), and PTSD (*β* = 0.39, *p* = 0.010), after accounting for potential confounders.

**Conclusion:**

The study found that individuals in the Kurdistan Region of Iraq who were exposed to climate-related stressors, such as extreme weather events and climate-induced homelessness, presented significantly poorer mental health outcomes compared to those without such exposure. These findings underscore the severe psychological impact of environmental changes and highlight the urgent need for targeted support within climate adaptation and disaster response strategies, especially for populations that are most directly affected.

## 1 Introduction

Exposure to climate-related events, such as drought, extreme heat, and flooding, increasingly contributes to adverse mental health outcomes, including elevated rates of psychological distress and increased psychiatric morbidity and mortality, particularly among disadvantaged individuals or those living in low-resource settings (1, 2). Rising temperatures and heat stress, along with other acute weather events, have been linked to a spectrum of mental health challenges, ranging from mild psychological distress to more severe mental health conditions, such as generalized anxiety disorder (GAD), major depressive disorder (MDD), posttraumatic stress disorder (PTSD), and, in the most extreme cases, suicide (3, 4). A systematic review and meta-analysis revealed that a 1 °C increase in temperature significantly contributed to increases in mental health-related morbidity and mortality (5). In view of this, and given that the climate is in constant flux, the consequences for mental health are no longer occasional or inconsequential, but pressing and persistent.

Specifically, individuals in high-risk environments, impoverished or displaced populations, and those with health disadvantages or poor healthcare access face the greatest mental health burden, even from moderate climate events (2, 6). The absence of adequate infrastructure and support systems, along with scarce financial resources, makes certain groups, particularly those in developing nations, highly vulnerable to adverse climate-related mental health effects. Evidence from Pakistan, for example, indicates that Pakistani children face significantly elevated mental health risks driven by climate-related anxiety (7, 8). Mortality rates from extreme weather events are up to 15 times greater in the most vulnerable regions compared to those least vulnerable (9). At the global level, these conditions are particularly pronounced in the MENA (Middle East and North Africa) region, including Iraq (10).

Ranked as the fifth most vulnerable country globally in terms of climate exposure, Iraq faces severe challenges from water scarcity, food insecurity, and extreme temperatures (11). According to the Iraqi Ministry of Environment, 39% of its land is already affected by desertification, with another 54% currently at risk (12). Within Iraq, the Kurdistan Region (KRI) has been increasingly impacted by frequent and intense extreme weather events (13). Projections indicate an expected temperature increase of 1.0 to 5.0 °C in Erbil, 1.0 to 6.0 °C in Sulaymaniyah, and 0.5 to 5.0 °C in Dohuk (13). Such weather shifts contribute to expanding dry lands, increasing sandstorms, progressing desertification, and exacerbating food and water insecurity (14).

Environmental changes increasingly trigger what is now referred to as climate anxiety and affect mental well-being both directly and indirectly (15). The direct effects may arise from witnessing environmental degradation and experiencing severe weather events. On a multidimensional level, mental health may be indirectly compromised through the ways in which climate change intersects with other systems and disrupts key determinants of psychological well-being, including working and living conditions, often resulting in displacement, food insecurity, loss of livelihoods, and economic insecurity (1, 16, 17). In the KRI, environmental stressors are increasingly disrupting daily life and traditional livelihoods, with 45% of rural residents reporting declines in crop production and fishing yields, along with losses of livestock (18). Further challenges arise from sociopolitical stressors such as poor water management, unsustainable agricultural practices, and transboundary water conflicts (19). These combined pressures appear to contribute to worsening mental health across the region. Recent evidence indicates that climate anxiety is more prevalent in the Kurdistan Region than central and southern Iraq (20).

Despite the region’s vulnerability, it remains underresearched in terms of climate-related hazards and their multifaceted impacts (12). While global recognition of the detrimental psychological impacts of climate change is continuing to increase, very limited scientific attention has been directed toward these issues in the Kurdistan Region of Iraq. This gap is likely related to insufficient institutional capacity, limited public awareness, and broader socioeconomic and political constraints that reduce the visibility and prioritization of climate-mental health research in the region. This is particularly concerning given the rise in climate-induced displacement, alongside other major displacement crises driven by political tensions and religious persecution, such as those affecting the Yazidi communities, adding an additional layer of psychosocial strain. A recent cross-sectional study conducted in the region revealed that most participants experienced moderate levels of eco-anxiety, with higher rates of severe anxiety reported in Erbil than in Sulaymaniyah and Duhok, especially among residents in urban areas and apartment settings (21). The combination of environmental degradation, urban stressors, and limited access to restorative green spaces underscores the complex socioecological determinants of climate-related mental health in the region and renders the KRI population increasingly vulnerable to psychological strains.

Yet, the limited body of existing research not only reflects a geographical gap but also reveals methodological shortcomings. Studies on the mental health impacts of climate change in the KRI are limited in scope and rely primarily on descriptive statistics and bivariate analyses, offering only a fragmented understanding of the complex interplay between environmental stressors and psychological outcomes (21, 22). These approaches fall short of capturing the multifactorial nature of climate-related mental health risks. Nevertheless, the initial findings point to a relevant and pressing issue underscoring the need for more robust, analytically grounded research that can deepen our understanding of these associations and guide effective interventions.

The present study offers the first substantial empirical evidence from this region by applying advanced statistical methods to analyse the impact of extreme weather events on psychological well-being. The use of multiple linear regression contributes to addressing this gap by offering a more rigorous examination of the relationships between climate-related stressors and mental health outcomes in this highly complex setting. Specifically, it investigates the extent to which climate-related factors influence or predict the mental health status of the population in the Kurdistan Region of Iraq and explores other individual-level and socioeconomic factors that may contribute to variations in climate-related mental health outcomes. By generating evidence-based insights from one of the countries most affected by climate change, this study aims to inform policy and health interventions, while contributing to the academic understanding of climate-related mental health risks in underrepresented contexts.

## 2 Materials and methods

### 2.1 Design and sampling

A cross-sectional study was conducted with a sample of 618 participants aged 18 years and older, residing in urban and rural areas of three governorates in the Kurdistan Region of Iraq: Duhok, Erbil, and Sulaymaniyah. An analytical cross-sectional approach was adopted as an early investigation to examine the impact of climate-related stressors on mental health and to assess the association between exposure to extreme weather events and mental health outcomes. This design allowed for a timely and comprehensive snapshot of these patterns and offered an initial evidence base for the Kurdistan Region of Iraq, where research on the mental health impacts of climate-related stressors remains limited. Moreover, logistical constraints, such as geographically dispersed communities and difficulties in maintaining consistent follow-up with participants, make longitudinal studies difficult to implement, further supporting the use of this approach.

We applied convenience sampling within predefined quotas to ensure balanced representation across the three governorates (Erbil, Sulaymaniyah, and Duhok) and between urban and rural areas. We set regional quotas to account for unequal access across governorates and to avoid over-representation of the most accessible region. Equal quotas of approximately 200 participants per governorate ensured equal representation across Erbil, Sulaymaniyah, and Duhok, preventing any single region from shaping the dataset. Given Erbil’s larger population compared to the other two regions, this approach maintained analytical balance and ensured that all regional contexts were equally reflected in the sample.

In each governorate, three trained data collectors were instructed to administer a total of 67 questionnaires (33 in urban areas and 34 in rural areas) to achieve a regionally balanced sample. Within these strata, eligible participants (residents aged 18 years and above) were approached and selected based on availability. Participants who were under 18 years of age or not residents of the designated governorates were excluded. Data collectors recruited participants in local public places and neighbourhoods recommended by the *Mukhtar* (Village head) of each area, as well as through house-to-house visits, particularly in rural settings. While participant selection was convenience-based, attention was given during data collection to achieving variation in gender, age, education level, and socioeconomic background.

### 2.2 Data collection tool

Our survey tool was designed to capture participants’ demographic information, exposure to extreme weather events, and psychological outcomes. The full questionnaire is provided as Supplementary material [see Additional file]. Occupational backgrounds as well as items capturing exposure to climate stressors were adapted from prior climate–health research (23). All instruments assessing mental health have been widely validated in international research and demonstrate good internal consistency, with Cronbach’s *α* values typically ranging between 0.88 and 0.94. We assessed a range of mental health constructs to define psychological distress using the following instruments:

#### 2.2.1 Patient health questionnaire (PHQ-8)

The PHQ-8 consists of 8 items on depressive symptoms over the past 2 weeks, rated on a 4-point Likert scale (0 = “not at all” to 3 = “nearly every day”), yielding a total score of 0–24 (minimal 0–4, mild 5–9, moderate 10–14, moderately severe 15–19, severe 20–24) (24). Excluding the suicidal ideation item of the PHQ-9, the PHQ-8 has been shown to be an effective tool for assessing depression in population-based studies, with an almost perfect correlation (r = 0.996) with the full scale (24, 25).

#### 2.2.2 Generalized anxiety disorder scale (GAD-7)

The GAD-7 comprises 7 items assessing generalized anxiety over the past 2 weeks, rated on the same 4-point Likert scale as the PHQ-8, yielding a total score of 0–21 (minimal 0–4, mild 5–9, moderate 10–14, severe 15–21) (26). It has excellent internal consistency (*α* = 0.92), good test–retest reliability, and robust procedural and convergent validity (26).

#### 2.2.3 Kessler psychological distress scale (K10)

The K10 includes 10 items measuring nonspecific distress over the past 4 weeks, on a 5-point scale (1 = “none of the time” to 5 = “all of the time”), resulting in a total score of 10–50 (low distress 10–19, mild distress 20–24, moderate distress 25–29, severe distress 30–50) (27). The K10 has shown strong psychometric properties, including effectiveness in distinguishing between DSM-IV cases and non-cases (28), and high internal consistency (*α* = 0.93) across different ethnic groups (29).

#### 2.2.4 PTSD checklist for DSM-5 (PCL-5, 8-item version)

The abbreviated PCL-5 comprises 8 items on PTSD symptoms in the past month, scored on a 5-point scale (0 = “not at all” to 4 = “extremely”). It has shown excellent reliability, with Cronbach’s α ranging from 0.90 to 0.91 in the original validation (30), and 0.90 to 0.93 in a subsequent systematic review (31).

#### 2.2.5 Climate change—man-made disaster-related distress scale (CC-MMDS)

The recently developed CC-MMDS contains 16 items rated on a 7-point scale, measuring climate distress over the last week. In our study, response options ranged from “strongly disagree” to “strongly agree.” After our data collection, the authors issued a corrigendum clarifying that intended labels were “does not apply at all” to “applies fully” (32). The measure has shown promising psychometric properties in the initial validation study, with Cronbach’s *α* = 0.94 and McDonald’s *ω* = 0.95 (33).

The questionnaire was translated into two Kurdish dialects, Sorani and Kurmanji, following forward and back-translation procedures conducted by professionals in the fields of psychotherapy and psychotraumatology practicing in the Kurdistan Region, ensuring linguistic accuracy and cultural appropriateness. The overall translation work was undertaken collaboratively by bilingual psychotherapy experts affiliated with the Institute of Psychotherapy and Psychotraumatology (IPP) in Duhok, in consultation with specialized medical professionals and academics, and was subsequently reviewed by two supervisors, one mental health specialist and one linguistics expert, who conducted a systematic quality assessment. The research team also held multiple meetings with the translators to refine terms and phrases, ensuring clarity and cultural relevance while accounting for linguistic nuances specific to the region.

The finalized questionnaire was made available online in three language options (Sorani, Kurmanji, and English), allowing participants to select their preferred language. Data were collected electronically using KoBo Toolbox, with survey administration supported by a trained research team based in the region. To ensure completeness of responses across all participants, all questions in the KoBo Toolbox survey were set as mandatory using the platform’s built-in mandatory-response feature. Data collectors, who were native speakers of the respective dialects, were trained to administer the survey and provide neutral clarifications when needed to ensure participants’ understanding of items or to explain complex terminology related to mental health or climate topics. When necessary, clarifications were also provided in Arabic to assist bilingual participants. Before launching data collection, test submissions were conducted to help data collectors identify any potential difficulties in administering the questionnaire, interpreting specific items, or using the online platform. During data analysis, internal consistency reliability was assessed for the PHQ-8, GAD-7, PCL-5, K10, and CC-MMDS instruments using Cronbach’s *α* to verify the reliability of the adapted scales in the study context.

### 2.3 Ethical considerations

Prior to participation, all the respondents were fully informed about the purpose and procedures of the study both verbally by trained researchers and via an integrated information sheet on the KoBo Toolbox platform, upon which they provided written informed consent. The study was conducted in accordance with institutional guidelines and the Declaration of Helsinki. Ethical approval was obtained from the University of Duhok, Iraq, on 23 February 2025 (reference number: 2309).

### 2.4 Sample size estimation

The required sample size was calculated on the basis of Cohen’s *f*^2^ effect size guidelines, using a power analysis conducted in R. Assuming a medium effect size (*R*^2^ = 0.13; *f*^2^ = 0.15), a statistical power of 80%, and a significance level of 0.05, the analysis indicated that a minimum of 117 participants was needed to detect the expected effect with sufficient power. Given that our final sample size of 618 far exceeds the minimum requirement, it provides power and reliability to detect even smaller effect sizes.

### 2.5 Statistical analysis

We applied quantitative methods, including significance testing, correlation analysis, and multiple linear regression modeling, to identify meaningful patterns. Multiple regression models were selected because they are appropriate for cross-sectional designs and suitable for early, field-based exploratory investigations aimed at generating initial evidence on emerging research areas. Recent studies from the region published during the review process have also employed similar regression-based approaches, underscoring the methodological relevance of this choice in comparable field contexts (20, 34). All statistical analyses were performed in RStudio (R version 4.4.3; released 28 February 2025) using the packages stats (35), pwr (36), and psych (37). Demographic characteristics (age, gender, education, occupation, region, area, and income) were summarized using frequencies and percentages (n, %) for categorical variables and mean, median, and range for continuous variables (Table 1).

**Table 1:** Demographic characteristics of the survey participants (*N* = 608).

Variable	Category	*N*	Percent
Age	Mean = 32.4; Sd = 11; Median = 30; Range = 18–75		
Gender	Male	314	51.6
	Female	294	48.4
Education	No formal education	47	7.7
	Primary	78	12.8
	Secondary	155	25.5
	Higher education	328	53.9
Occupation	Farmer	46	7.6
	Day laborer	85	14.0
	Small/Medium Business	47	7.7
	Housewife	95	15.6
	Unemployed	72	11.8
	Academic/Researcher	66	10.9
	Healthcare Professional	54	8.9
	Other	143	23.5
Region	Duhok	200	32.9
	Erbil	206	33.9
	Sulaymaniyah	202	33.2
Area	Rural	311	51.2
	Urban	297	48.8
Income	<500,000 IQD	186	30.6
	500,000–1,000,000 IQD	267	43.9
	1,000,001–2,000,000 IQD	115	18.9
	>2,000,000 IQD	40	6.6

The table presents the demographic characteristics of participants (*N* = 608) from the Kurdistan Region of Iraq. Age is reported as mean, standard deviation, median, and range. *n*, number of participants; Percent, percentage of total sample.

Group comparisons (Table 2) were conducted using t-tests for variables with two categories (e.g., gender/area), and one-way ANOVA for variables with more than two categories (e.g., region). Normality of outcome scores was assessed with Shapiro–Wilk tests and visual inspection of histograms. Levene’s test for homogeneity of variance was conducted for each mental health outcome across the grouping variable “region” prior to ANOVA analyses. The assumption of equal variances was met (all *p*-values > 0.05).

**Table 2:** Group comparisons of mental health scores by sociodemographic and climate-related variables (*N* = 608).

Variable	Group	PHQ-8		GAD-7		K-10		CC-MMDS		PCL-5	
		Summary	*P*	Summary	*P*	Summary	*P*	Summary	*P*	Summary	*P*
Gender	Male	8.28 ± 5.21	0.026*	6.76 ± 4.26	0.064	24.42 ± 8.26	0.024*	53.71 ± 22.22	0.830	8.7 ± 7.86	0.002*
	Female	9.19 ± 4.87		7.44 ± 4.62		25.94 ± 8.29		53.31 ± 23.98		10.67 ± 7.75	
Area	Rural	8.24 ± 4.67	0.016*	6.88 ± 4.06	0.233	24.33 ± 7.78	0.013*	53.67 ± 21.29	0.867	9.06 ± 7.27	0.058
	Urban	9.23 ± 5.4		7.31 ± 4.81		26.01 ± 8.75		53.36 ± 24.83		10.27 ± 8.41	
Region	Duhok	8.59 ± 5.03	0.838	6.06 ± 4.19	0.000*	23.44 ± 8.29	0.000*	43.35 ± 22.32	0.000*	7.93 ± 7.74	0.000*
	Erbil	8.88 ± 5.19		7.68 ± 4.69		26.82 ± 8.68		61.5 ± 23.01		11.92 ± 7.7	
	Sulaymaniyah	8.69 ± 4.98		7.51 ± 4.27		25.15 ± 7.57		55.44 ± 20.04		9.04 ± 7.65	
Employed	Yes	8.48 ± 5.21	0.040*	7.07 ± 4.41	0.885	25.02 ± 8.33	0.541	55.25 ± 23.26	0.002*	9.69 ± 8	0.836
	No	9.37 ± 4.59		7.13 ± 4.54		25.49 ± 8.25		48.94 ± 21.96		9.54 ± 7.51	
C. Home-lessness	Yes	10.63 ± 4.23	0.003*	8.8 ± 5.03	0.019*	27.61 ± 7.59	0.027*	58.8 ± 22.36	0.102	12.7 ± 8.46	0.014*
	No	8.57 ± 5.1		6.95 ± 4.37		24.95 ± 8.33		53.09 ± 23.09		9.4 ± 7.77	
C. Events	Yes	9 ± 5.01	0.043*	7.26 ± 4.42	0.154	25.55 ± 8.17	0.086	54.62 ± 23.3	0.074	10.38 ± 7.85	0.001*
	No	8.1 ± 5.14		6.7 ± 4.49		24.27 ± 8.55		51.05 ± 22.42		8.02 ± 7.68	

The table summarizes the means and standard deviations of the total scores for five mental health measures (PHQ-8, GAD-7, PCL-5, K-10, and CC-MMDS) across key sociodemographic and climate factors in the Kurdistan Region of Iraq. Summary values are presented as the means ± standard deviations (M ± SD). Reported *p*-values reflect the significance of group differences tested using independent *t*-tests for binary variables (all except region) and one-way ANOVA for variables with more than two categories (region). Bold values with an asterisk (*) indicate statistically significant results (*p* < 0.05). C. Homelessness = climate-related homelessness; C. Events = climate-related extreme events.

We conducted a series of multiple linear regression analyses (Table 3) to estimate the effects of demographic variables, socioeconomic status (SES), geographic factors, and climate-related stressors on five mental health outcomes: PHQ-8, GAD-7, K10, PCL-5, and CC-MMDS. The total scores for mental health measures were z-standardized (*M* = 0, SD = 1) prior to analysis, enabling direct comparison of regression coefficients across models. We evaluated all candidate models using standard fit statistics, including the overall F-test p-value, AIC, and Adjusted *R*^2^. For the final selection, we prioritised Adjusted *R*^2^ and AIC, as together they provide robust indicators of explanatory power while appropriately penalising unnecessary model complexity. The model with the highest Adjusted *R*^2^ and lowest AIC was therefore selected as the most appropriate. After selecting the final model, standard diagnostic checks as well as multicollinearity tests were performed to verify that the assumption of linear regression was met. The normality assumption was assessed by visually inspecting residuals using Q–Q plots.

**Table 3:** Regression results for all mental health outcomes.

*N* = 608	PHQ-8^a^		GAD-7^a^		K10^a^		PCL-5^a^		CC-MMDS^a^	
Predictor	β [95%-CI]	*P*	β [95%-CI]	*P*	β [95%-CI]	*P*	β [95%-CI]	*P*	β [95%-CI]	*P*
(Intercept)	−0.18 [−0.57–0.20]	0.350	−0.48 [−0.87–−0.10]	0.013*	−0.50 [−0.88–−0.11]	0.011*	−0.51 [−0.88–−0.13]	0.008*	−0.96 [−1.32–−0.60]	<0.001*
Age	−0.01 [−0.01–0.00]	0.183	0.00 [−0.01–0.01]	0.623	0.00 [−0.01–0.01]	0.574	0.00 [−0.01–0.01]	0.879	0.01 [0.01–0.02]	<0.001*
Female	0.11 [−0.07–0.29]	0.226	0.10 [−0.07–0.28]	0.251	0.12 [−0.06–0.30]	0.190	0.24 [0.06–0.41]	0.008*	−0.00 [−0.17–0.16]	0.963
No higher education	−0.05 [−0.23–0.12]	0.554	−0.11 [−0.28–0.07]	0.221	−0.11 [−0.29–0.06]	0.203	−0.08 [−0.25–0.09]	0.376	−0.13 [−0.29–0.04]	0.123
Unemployed	0.11 [−0.10–0.32]	0.289	−0.02 [−0.23–0.19]	0.846	0.01 [−0.20–0.22]	0.924	−0.15 [−0.36–0.05]	0.144	−0.27 [−0.47–−0.07]	0.007*
Lower income	0.11 [−0.08–0.30]	0.249	0.11 [−0.08–0.30]	0.248	0.07 [−0.12–0.26]	0.469	0.04 [−0.15–0.22]	0.694	0.15 [−0.03–0.33]	0.104
Urban	0.18 [0.01–0.34]	0.039*	0.07 [−0.10–0.23]	0.441	0.18 [0.01–0.34]	0.037*	0.11 [−0.05–0.28]	0.175	−0.02 [−0.18–0.14]	0.794
Region										
Erbil	0.03 [−0.17–0.23]	0.763	0.37 [0.17–0.57]	<0.001*	0.39 [0.19–0.58]	<0.001*	0.46 [0.26–0.65]	<0.001*	0.83 [0.64–1.02]	<0.001*
Sulaymaniyah	−0.01 [−0.22–0.19]	0.895	0.33 [0.13–0.53]	0.001*	0.18 [−0.02–0.38]	0.079	0.08 [−0.12–0.27]	0.440	0.56 [0.37–0.75]	<0.001*
Experienced C. Events	0.13 [−0.05–0.30]	0.150	0.07 [−0.11–0.24]	0.454	0.09 [−0.08–0.26]	0.307	0.23 [0.06–0.40]	0.008*	0.11 [−0.05–0.28]	0.181
Experienced C. Homelessness	0.38 [0.08–0.69]	0.014*	0.42 [0.11–0.72]	0.007*	0.32 [0.01–0.62]	0.042*	0.39 [0.09–0.69]	0.010*	0.25 [−0.04–0.54]	0.087
*R*^2^/*R*^2^ adjusted	0.040/0.024		0.049/0.033		0.053/0.037		0.090/0.074		0.152/0.137	

a Total score of the outcome variable was standardized (*z*-score) prior to analysis.

The table presents estimates (β), confidence intervals (CI), and *p*-values from the chosen multiple regression model (Model 4) for all mental health outcomes. Bold values with an asterisk (*) indicate statistically significant results (*p* < 0.05). Predictors include demographic variables, socioeconomic status, geographic factors, and climate-related stressors. The dummy variables were coded as follows: gender (0 = male, 1 = female); higher education (0 = yes, 1 = no); employment (0 = employed, 1 = unemployed); income (0 = higher (defined as >1,000,000 IQD), 1 = lower); region (0 = Duhok, 1 = Erbil) and (0 = Duhok, 1 = Sulaymaniyah); area (0 = rural, 1 = urban); climate-related events (0 = no, 1 = yes); and climate-related homelessness (0 = no, 1 = yes).

**Figure 1:**
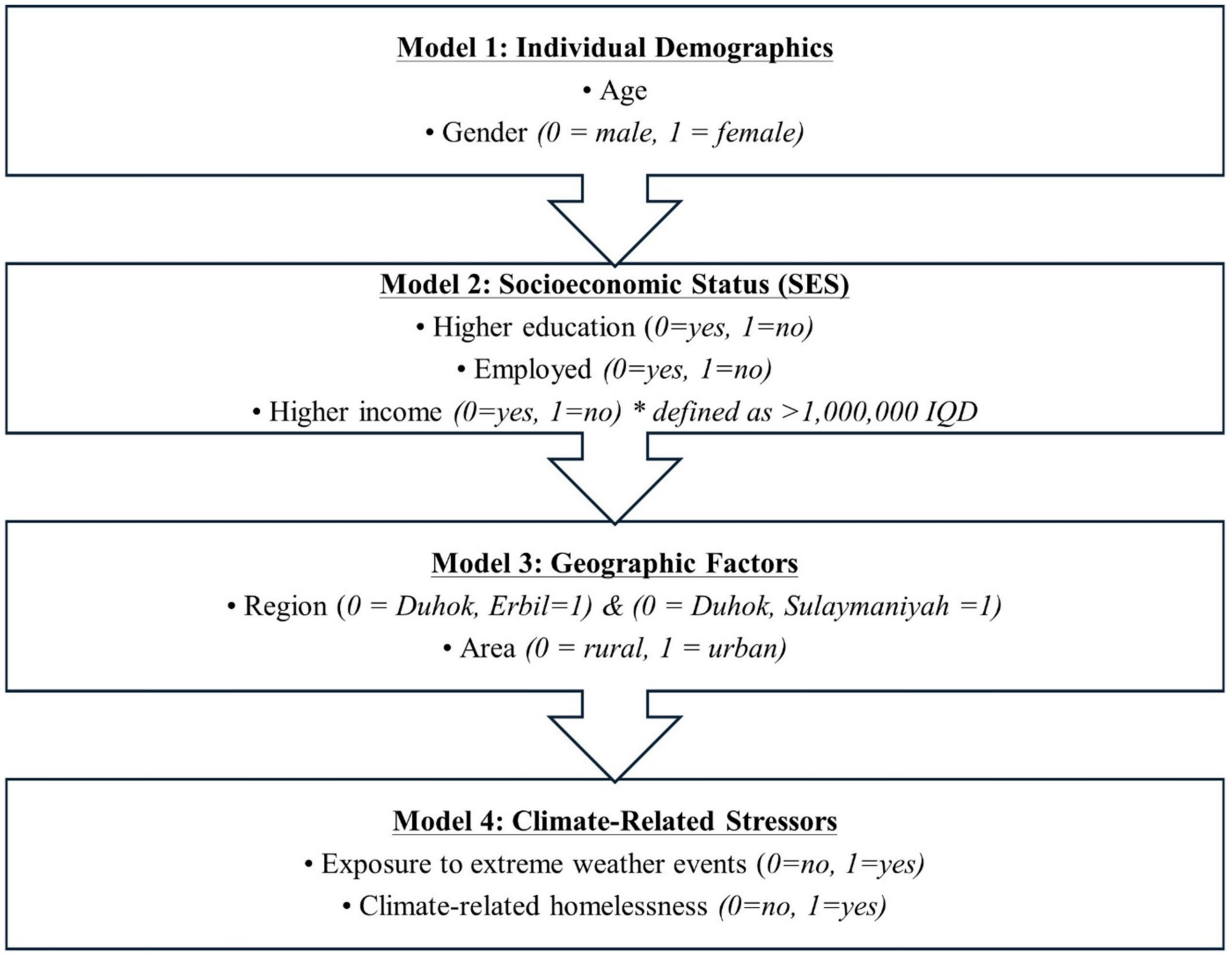
Block entry process in the hierarchical blockwise regression. The figure illustrates the inclusion of variable blocks in the hierarchical blockwise regression analyses conducted for each mental health outcome (PHQ-9, GAD-7, K10, PCL-5, and CC-MMDS). The model-building process progressed from individual-level variables to socioeconomic, geographic, and climate-related predictors.

A hierarchical blockwise regression approach was applied for each mental health outcome, beginning with Model 0 (intercept-only) and sequentially adding conceptually grouped blocks of predictors, as illustrated in Figure 1. The sequence of blocks reflected the assumption that factors more closely related to the individual would have a stronger influence on mental health than broader contextual variables. Accordingly, Model 1 included individual demographics (age and gender). Model 2 added socioeconomic status variables (income, education, and employment). Model 3 incorporated geographic factors (area and region). Finally, Model 4 introduced the main predictors of interest—climate-related variables—specifically, exposure to extreme weather events and climate-related homelessness. The choice of hierarchical blockwise regression was made because it allows for the systematic assessment of predictors in conceptually meaningful steps, thereby clarifying the incremental contribution of climate-related factors beyond individual, socioeconomic, and geographic variables. This approach is particularly suitable for exploratory studies in underresearched regions, where multiple layers of determinants interact. However, hierarchical blockwise regression also has limitations. The order of variable entry, while theoretically justified, can influence results and may not fully capture reciprocal or complex relationships among predictors. Moreover, the method does not account for potential latent variables or measurement error, as would be possible in structural equation modeling (SEM). Because the analysis is based on cross-sectional data collected at a single time point, it cannot assess temporal dynamics or establish causal pathways. Thus, while hierarchical blockwise regression offers transparency and comparability, the findings should be interpreted with caution, and future studies may benefit from applying more advanced multivariate approaches such as SEM or longitudinal designs to better capture causal pathways and temporal dynamics in climate-related mental health outcomes.

## 3 Results

### 3.1 Characteristics of the survey sample

A total of 618 participants were surveyed, and 10 were excluded during data cleaning due to implausible age-related responses, missing consent, or being under the age of 18. The final analyzed sample consisted of 608 participants with a balanced gender distribution: 314 males (51.6%) and 294 females (48.4%). Participants’ ages ranged from 18 to 75 years (*M* = 32.4, SD = 11), with the interquartile range indicating that 50% of participants were aged between 24 and 39 years. More than half of the sample (53.9%) held a higher education degree, followed by 25.5% with secondary education, 12.8% with primary education, and 7.7% with no formal education. In terms of regional representation, participants were distributed across Duhok (32.9%), Erbil (33.9%), and Sulaymaniyah (33.2%), with each region comprising both rural and urban residents. Occupational backgrounds varied and included farmers (7.6%), day laborers (14%), small/medium business owners (7.7%), housewives (15.6%), unemployed individuals (11.8%), academics/researchers (10.9%), healthcare professionals (8.9%), and others (23.5%). Furthermore, the most prevalent income level was between 500,000 and 1,000,000 IQD, reported by 43.9% of the sample. A detailed summary of the sample’s demographic characteristics is presented in Table 1.

### 3.2 Extreme weather events

**Figure 2:**
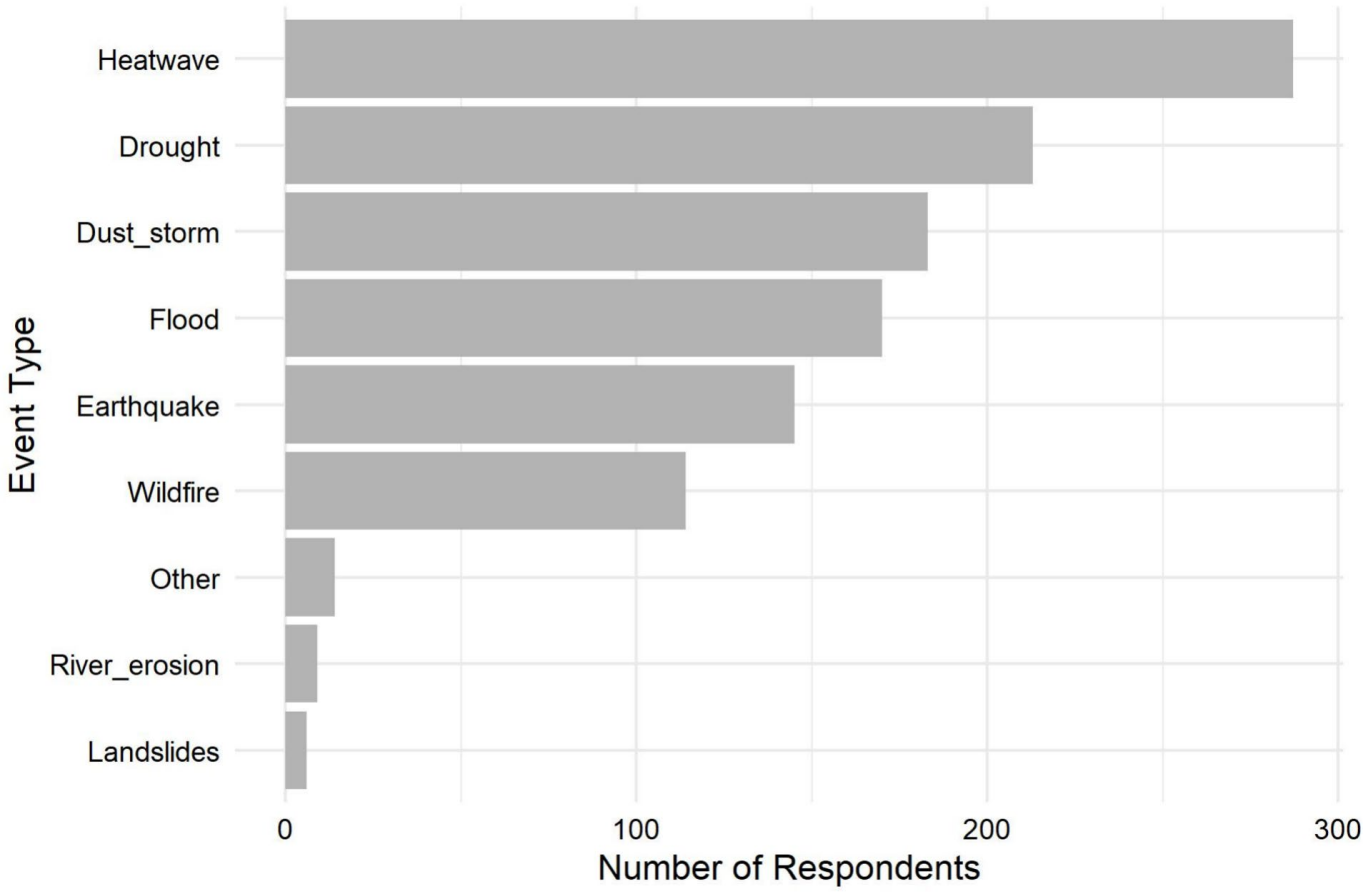
Frequency of exposure to extreme weather in the Kurdistan Region of Iraq. Participants (*N* = 608) responded to a multiple-response question about extreme weather events they had experienced while living in the region. The figure shows how often each event was reported.

The most commonly reported extreme weather events-from most to least frequent-were heatwaves, droughts, and dust storms, followed by floods, earthquakes, and wildfires (see Figure 2). Fewer respondents reported experiencing landslides and riverbank erosion. Because participants could report multiple events, the total frequency across all event types did not equal the number of individuals surveyed.

### 3.3 Mental health measures

Cronbach’s alpha coefficients were calculated for each scale to assess internal consistency reliability and to examine how well the translated items worked together in our sample. The PHQ-8, GAD-7, K10, PCL-5, and CC-MMDS demonstrated good to excellent reliability, with *α* values of 0.88, 0.88, 0.92, 0.93, and 0.95, respectively (values reported to two decimal places). These results suggest that the translated Kurdish versions functioned reliably within the study sample, although formal psychometric validation has not yet been conducted.

As for the total scores of the mental health measures, the mean PHQ-8 score in the sample was 8.7 (SD = 5.1), with values ranging from 0 to 24, indicating mild depressive symptoms among the participants based on PHQ-8 cutoffs (minimal 0–4, mild 5–9, moderate 10–14, moderately severe 15–19, severe 20–24). The average GAD score was 7.1 (SD = 4.4), spanning the full scale range from 0 to 21, which aligns with mild anxiety as per the established clinical cutoffs (minimal 0–4, mild 5–9, moderate 10–14, severe 15–21). Scores on the K10 averaged 25.2 (SD = 8.3), with responses covering the entire scale range from 10 to 50. For the abbreviated 8-item PCL-5, the mean score was 9.6 (SD = 7.9). A small difference between the mean and median suggested mild skewness, likely due to a subset of participants reporting elevated levels of posttraumatic stress symptoms. Finally, the mean score on the CC-MMDS was 53.5 (SD = 23.1), with values ranging from 16 to 112. The range of responses for all five scales, covering the full span of each measure, reflects a diverse sample and a wide distribution of symptom severity.

### 3.4 Group differences in mean mental health scores

Table 2 presents all group comparisons described below.

#### 3.4.1 Gender

Statistically significant gender differences were observed in the PHQ-8, K10 and PCL-5 scores. Female participants reported higher scores on the PHQ-8 (*M* = 9.19, SD = 4.87) compared to males (*M* = 8.28, SD = 5.21), reflecting greater depressive symptoms. Similarly, women scored higher on the K10 (*M* = 25.94, SD = 8.29) than men did (*M* = 24.42, SD = 8.26). A similar trend was found on the PCL-5, with females reporting greater PTSD symptoms (*M* = 10.67, SD = 7.75) than males did (*M* = 8.7, SD = 7.86).

#### 3.4.2 Area (urban/rural)

Participants from urban areas reported significantly higher mean scores on the PHQ-8 (*M* = 9.23, SD = 5.4) than did those from rural areas (*M* = 8.24, SD = 4.67). A similar pattern was observed for the K10, with higher scores among participants from urbanized areas (*M* = 26.01, SD = 8.75) than among participants living in rural settings (*M* = 24.33, SD = 7.78).

#### 3.4.3 Region (Erbil/Duhok/Sulaymaniyah)

ANOVA revealed a significant effect of region on the GAD-7, K10, PCL-5, and CC-MMDS scores. Across all four scales, a consistent trend was observed: participants from Erbil reported the highest scores (e.g., GAD-7: *M* = 7.68, SD = 4.69), followed by those from Sulaymaniyah (e.g., GAD-7: *M* = 7.51, SD = 4.27), whereas participants from Duhok reported the lowest scores (e.g., GAD-7: *M* = 6.06, SD = 4.19). Similar patterns were found for the K10, PCL-5, and CC-MMDS.

#### 3.4.4 Socioeconomic status (education, income, employment)

No statistically significant group differences were detected between individuals with and without higher education on any of the measures. Similarly, having a higher income (>1,000,000 IQD) did not significantly affect scores. Employment status, however, was significant on the PHQ-8, with unemployed individuals scoring higher (*M* = 9.37, SD = 4.59) than employed individuals (*M* = 8.48, SD = 5.21). Conversely, employed participants reported higher CC-MMDS scores (*M* = 55.25, SD = 23.26) than unemployed participants did (*M* = 48.94, SD = 21.96).

#### 3.4.5 Climate change-related stressors (climate change-related homelessness, exposure to extreme weather events)

Climate-related homelessness was associated with significantly higher scores on the PHQ-8, GAD-7, K10, and PCL-5. For example, homeless participants scored higher on the PHQ-8 (*M* = 10.63, SD = 4.23) than non-homeless participants did (*M* = 8.57, SD = 5.1). Exposure to extreme weather events was associated with higher scores on the PHQ-8 (*M* = 9, SD = 5.01) compared to no exposure (*M* = 8.1, SD = 5.14) and on the PCL-5 (*M* = 10.38, SD = 7.85) relative to those unexposed (*M =* 8.02, SD = 7.68).

### 3.5 Multiple linear regression

The results indicated that Model 4, which included climate-related stressors, yielded the highest adjusted *R*^2^ across all measures: PHQ-8 (*R*^2^ = [0.024]), GAD-7 (*R*^2^ = [0.033]), K10 (*R*^2^ = [0.037]), PCL-5 (*R*^2^ = [0.074]), and CC-MMDS (*R*^2^ = [0.137]), suggesting that the inclusion of climate predicators significantly improved the explanatory power of the model and contributed meaningfully to the variation in the mental health outcome variables. As such, Model 4 was chosen as the final model to predict depression, anxiety, psychological distress, PTSD, and climate-related distress outcomes, as presented in detail below (Table 3). All the coefficients represent differences in the outcomes expressed in standard deviation units.

#### 3.5.1 Demographic predicators

Age was a significant positive predictor of the CC-MMDS (*β* = 0.01, *p* < 0.001), meaning that the climate-related distress score increased with each additional year of age. Female gender demonstrated a meaningful positive association with PCL-5 scores (*β* = 0.24, *p* = 0.008) relative to men.

#### 3.5.2 Socioeconomic status (SES) predictors

Higher education attainment and income level showed no significant results across all the models. Unemployment, on the other hand, was a significant negative predictor in the CC-MMDS model (*β* = −0.27, *p* = 0.007), pointing to being employed as associated with greater climate-related distress levels.

#### 3.5.3 Geographical predictors

Participants living in urban areas had higher scores on both the PHQ-8 (*β* = 0.18, *p* = 0.039) and K10 (*β* = 0.18, *p* = 0.037) than did those residing in rural settings.

Individuals living in Erbil experienced greater psychological strain, as reflected by higher GAD-7 (*β* = 0.37, p < 0.001), K10 (*β* = 0.39, p < 0.001), PCL-5 (*β* = 0.46, p < 0.001), and CC-MMDS (*β* = 0.83, p < 0.001) scores, relative to those in Duhok. In a similar pattern, residents of Sulaymaniyah reported significantly higher scores on the GAD-7 (*β* = 0.33, *p* = 0.001) and CC-MMDS (*β* = 0.56, p = 0.001) than individuals from Duhok did.

#### 3.5.4 Climate-related predictors

Having experienced extreme weather events showed a significant positive effect on the PCL-5 score (*β* = 0.23, *p* = 0.008), underscoring that exposure to extreme weather events is linked to exhibiting posttraumatic symptoms. Additionally, climate-related homelessness functioned as a significant predictor and was associated with increased scores across four measures: the PHQ-8 (*β* = 0.38, *p* = 0.014), GAD-7 (*β* = 0.42, *p* = 0.007), K10 (*β* = 0.32, *p* = 0.042), and PCL-5 (*β* = 0.39, *p* = 0.010).

## 4 Discussion

### 4.1 Findings

This study set out to investigate the mental health impacts of climate change-related stressors in the Kurdistan Region of Iraq (KRI), a region that remains underrepresented in the global literature on climate-related mental health. Against the backdrop of increasing environmental degradation, sociopolitical stressors, and rising temperatures in the region, we aimed to assess how exposure to extreme weather events predicts mental health outcomes among KRI residents. In contrast to previous studies in the KRI, which have primarily employed descriptive and bivariate approaches (e.g., t-tests and chi-square tests) to examine differences in mental health outcomes across demographic groups, our study combined bivariate analyses with a regression modeling approach. This allowed us not only to explore group-level differences but also to control for multiple demographic and contextual factors, providing deeper insights into the complex relationships between climate-related exposures and mental health outcomes.

Our findings show that exposure to extreme weather events was significantly associated with elevated posttraumatic stress symptoms. Participants with higher levels of climate-related distress were also more likely to report elevated symptoms across multiple mental health domains. These results align with those of prior studies demonstrating that environmental disasters and extreme weather events can trigger lasting psychological trauma rather than short-term distress, increasing the risk for PTSD and other mental health disorders (4). Comparable results were reported after Hurricane Katrina in the United States, where long-term PTSD rates remained elevated years after the disaster (38), and in Bangladesh, where climate-related flooding was associated with higher levels of depression and anxiety (39). Together, these findings underscore the profound and multifaceted mental health burden that direct climate impacts can impose on affected populations.

The degree to which climate change impacts mental health depends largely on the severity of exposure and the nature of its consequences. Previous research has shown that individuals directly affected by climate-related events—such as those experiencing displacement, livelihood loss, or personal harm—are particularly vulnerable to psychological distress (17). A study from Iraq highlighted these disparities, demonstrating a disproportionate psychological impact of extreme weather events on disadvantaged populations, namely, displaced populations (22). Consistent with these findings, in our study climate-related homelessness stood out as a clear marker of this heightened vulnerability, with displaced participants reporting significantly higher levels of depression, anxiety, PTSD symptoms, and psychological distress than those who did not experience such displacement. This pattern highlights displacement as a critical pathway through which climate stressors translate into enduring mental health challenges. Comparable evidence has been documented in Sub-Saharan Africa and South Asia, where climate-induced migration is consistently linked to higher levels of distress and suicidal ideation (40).

In our study, heatwaves, droughts, and dust storms were the most frequently reported climate-related events, aligning with those identified as most prevalent in the International Organization for Migration (IOM) report on climate vulnerability in the Kurdistan Region of Iraq (18). These events are not experienced in the same way across populations; rather, different types of extreme weather shape vulnerability in distinct ways. Although we did not examine group-specific disparities or investigate how each event affects different populations, previous research provides important insight into these patterns. For example, farmers are often disproportionately affected by drought because reductions in agricultural yield directly threaten their livelihoods and economic stability (3, 18). Evidence from Iran and Ghana similarly shows that drought-related stressors impose a substantial mental health burden on farmers (41, 42). Meanwhile, displaced households, individuals living in poor housing conditions, outdoor workers, pregnant women, children, and older adults face greater health risks during heatwaves because their living conditions, work environments, or physiological characteristics reduce their ability to avoid or withstand extreme heat (3, 5, 43–45). Dust storms, in contrast, disproportionately impact individuals with pre-existing chronic conditions, particularly cardiovascular and pulmonary diseases, who are more susceptible to respiratory and physiological stress (3, 46). Taken together, this evidence suggests that the most frequently reported climate-related events may contribute to climate-related distress in the region through differentiated pathways across population groups. Future research in the Kurdistan Region would therefore benefit from examining how specific events affect distinct groups through both direct mechanisms (e.g., heat stress, physical discomfort) and indirect mechanisms (e.g., livelihood loss, displacement, economic strain).

Age was a significant positive predictor of climate-related distress, with scores on climate-related distress increasing with each additional year. This pattern may indicate a cumulative psychological burden, where repeated exposure to environmental degradation over time heightens perceived vulnerability. Alternatively, older individuals may possess a greater awareness of long-term ecological changes and their implications, leading to stronger emotional responses. The higher distress levels among older adults may also be explained by their increased physical vulnerability to climate-related hazards, including greater susceptibility to heat stress, the heavier toll such events take on physical functioning, and the coexistence of chronic health conditions that can exacerbate climate impacts. Similarly, prior studies have identified older age as a factor that increases vulnerability to elevated temperature–related morbidity and mortality (5). Emerging evidence from the region has also shown that older adults exhibit higher levels of climate anxiety (34). In this context, our findings suggest that age-related differences in climate distress may reflect both lived experience and heightened risk perception. Future research should aim to disentangle the relative contributions of these factors to better understand the mechanisms driving age-related differences in climate-related distress.

Employment status also emerged as a significant factor, with employed participants reporting higher levels of climate-related distress than their unemployed counterparts. This finding is somewhat counterintuitive, as unemployment is often associated with poorer mental health. One possible explanation is that individuals engaged in livelihoods sensitive to climate variability such as farming, small-scale trade, or other resource-dependent sectors may experience heightened distress about future income stability. Additionally, the employed often face greater exposure to heat and high ambient temperatures, as in the case of fieldwork under sun exposure or in the context of frequent electricity shortages in the workplace, resulting in workers enduring poorly conditioned environments. This pattern is comparable to findings from studies in Southern Europe, where farmers and construction workers reported significantly higher eco-anxiety and stress levels than unemployed respondents (47). Another possibility is that employed individuals, by virtue of their economic engagement and information access, may be more aware of climate risks and their potential consequences. This interpretation resonates with the growing recognition of climate or ecological anxiety, a phenomenon characterized by negative emotions such as worry, fear, and helplessness in response to climate change and its associated threats (48, 49). Thus, rather than indicating better resilience, higher climate distress among employed individuals may reflect the intersection of occupational vulnerability and heightened environmental awareness.

Additionally, our analysis revealed notable regional disparities in mental health outcomes. Specifically, individuals in both Erbil and Sulaymaniyah presented higher levels of climate-related distress than did those in Duhok. This pattern aligns with earlier findings from Mohammed (21) documenting greater eco-anxiety in Erbil than in the other two governorates. In the KRI, this may reflect the particular environmental pressures facing major cities such as Erbil, where vegetation cover has declined by over 50% and surface water resources have decreased by over 41% (50). Climate-related conditions vary across these three governorates, with IOM data revealing more pronounced increases in temperature and drought in several districts of Erbil and Sulaymaniyah compared to Duhok. This pattern is further reflected in the disproportionately greater decline in irrigation water, along with more severe effects on livelihoods and larger losses in crop production, relative to Duhok (18). These trends help explain the heightened climate-related distress observed in Erbil and Sulaymaniyah in our study.

Moreover, regional differences in climate-related distress may reflect broader demographic and contextual contrasts across the three governorates. Erbil, the political and economic capital of KRI, is the largest urban center (≈14,471 km^2^) and has a large and diverse population shaped by migration and displacement (51). Sulaymaniyah is likewise highly urbanized (≈13,368 km^2^) (51), whereas Duhok is smaller and more rural–mountainous (≈ 10,955.91 km^2^) (52). Erbil has experienced rapid population growth, displacement, and migration driven by regional political instability, including displacement resulting from the Islamic State and the Syrian crisis. Notably, Erbil alone has hosted nearly half of all refugees in Iraq (47.9%) (53). Consistent with these structural differences, the 2024 census reports higher population concentrations in Erbil (2,517,534) and Sulaymaniyah (2,401,724) than in Duhok (1,599,871) (54). Such population pressures exacerbate existing climate challenges by increasing demand on natural resources, water supplies, infrastructure, and electricity, thereby amplifying stress on affected communities. Given Erbil’s role as the region’s central urban hub, and in contrast to Duhok’s more rural context, the differences observed in our findings could be attributed to stressors typically associated with urban environments, such as economic pressures, work-related stress, industrial pollution, environmental degradation, and limited access to green spaces. Environmental stressors, alongside broader urban and socioeconomic pressures, may have contributed to the elevated distress levels observed in our data. The underlying mechanisms highlight that the climate–mental health relationship extends beyond direct climate impacts, reflecting a more complex interplay between environmental and sociopolitical conditions.

It is also worth mentioning that beyond climate-related distress, the study revealed notable regional differences in non-climate-specific mental health outcomes, as predicted by the climate-adjusted models. Similarly, poorer mental health outcomes were found among individuals in Erbil and Sulaymaniyah than among those in Duhok. These results are in line with patterns observed in earlier research from the region that examined mental health irrespective of climate influences. For example, in the context of COVID-19, one study on survivors reported similar trends, with those in Erbil and Sulaymaniyah showing higher levels of stress, anxiety, and depression than those in Duhok (55). Moreover, disparities emerged between urban and rural areas, with urban residents reporting significantly higher levels of depression and overall psychological distress. The identified regional and urban–rural differences in the climate-adjusted models suggest that mental health outcomes in the KRI are shaped not only by direct climate impacts but also by structural, socioeconomic, and contextual conditions that shape vulnerability.

This study addresses a critical research gap in the Global South, particularly in regions highly exposed to climate change but underrepresented in mental health research. By combining bivariate analyses with multivariate regression modeling, our approach moves beyond descriptive associations to account for multiple demographic, geographic, and climate-related predictors simultaneously. This methodological rigor enables us to more confidently isolate the relevance and influence of climate-related factors on mental health, even in settings where numerous overlapping stressors are present. In doing so, the study demonstrates the value of integrating multiple contextual layers when assessing psychological risk in severely affected regions.

Taken together, our findings show that climate change in the Kurdistan Region of Iraq is already entangled with patterns of psychological distress, especially among climate-displaced persons, older adults, workers in climate-sensitive sectors, and residents of rapidly transforming urban environments. Ignoring these mental-health impacts risks deepening existing inequalities and undermining the effectiveness of climate adaptation as a whole. Mental health must therefore be treated as a core component of climate policy in the KRI rather than a secondary concern. In practical terms, this requires integrating psychosocial support into climate-related displacement and disaster-response systems, for example through brief distress screening, on-site counselling in temporary shelters, and mental-health components within drought, flood, and heat-response plans. Coordination between health services, disaster-relief actors, and climate-policy units is essential, including shared procedures, referral pathways, and early-warning indicators for mental health. International organisations and donors can contribute by aligning climate financing with mental-health priorities, building frontline capacity, and supporting scalable, low-threshold community programmes. Evidence from international intervention models, such as the Good Grief Network, the Work That Reconnects, or group-based climate-distress programs, shows that community-based formats (peer support groups, facilitated workshops, psychoeducation, online journaling, structured group sessions) can effectively reduce eco-distress and improve coping (56). While most were developed in high-income contexts, their structure, emphasis on collective processing, and focus on meaning-making align closely with coping preferences documented in the KRI, where communal, spiritual, and narrative-based strategies are central. Adapting these models to the Kurdish context represents a feasible and culturally congruent approach, especially given the low acceptability of clinic-based mental-health care in many parts of the region. This is further underscored by findings from Kizilhan & Weigelt, who show that survivors overwhelmingly rely on spiritual and communal coping, while formal mental-health services are often perceived as least helpful (57). Their work highlights the importance of culturally grounded psychosocial care that integrates spirituality, communal narratives, justice frameworks, and collective meaning-making. Researchers and practitioners in the Kurdistan Region should therefore co-design and rigorously evaluate interventions that bridge evidence-based psychosocial methods with culturally meaningful communal and spiritual practices.

### 4.2 Limitations

Some of the tools used to assess mental health outcomes, particularly the CC-MMDS scale, were originally developed for Western populations and may not be fully culturally appropriate for capturing the subjective experiences of psychological distress in the Kurdish context. Here, ongoing sociopolitical and economic struggles can easily overshadow concerns about climate change, which may lead participants to underreport or to attribute less importance to these feelings when answering the questions. Additionally, due to limited internet access and varying levels of literacy among participants, the questionnaire was not entirely self-administered. Instead, support was provided by trained members of the research team, which may have influenced participants’ responses and introduced a degree of social desirability bias.

Although we observed higher psychological distress and climate-related distress scores in Erbil and Sulaymaniyah than in Duhok, the three regions (Erbil/Duhok/Sulaymaniyah) differ in levels of urbanization, population dynamics, migration and displacement patterns, and other socioeconomic factors. Hence, these mental health differences cannot be interpreted as solely reflecting climate exposure and may also represent other contextual stressors and broader urban, demographic, and sociopolitical pressures in addition to climate factors. Given the cross-sectional design and the complexity of these contextual variations, causal inferences are limited. Future research should examine these mechanisms more explicitly, for example through longitudinal designs and structural equation modeling (SEM). Stronger designs and modeling approaches would help disentangle climate-specific effects from other regional drivers of distress more confidently.

## 5 Conclusion

The study revealed that individuals in the Kurdistan Region of Iraq who were exposed to climate-related stressors, defined by extreme weather events and climate-induced homelessness, exhibited significantly poorer mental health outcomes than those reporting no such exposure. These findings underscore the profound psychological impact of environmental changes and highlight the urgent need for targeted support within climate adaptation and disaster response strategies, especially for populations that are most directly affected. They also point to the importance of applying more complex extensions of multivariate analyses, as well as longitudinal designs and qualitative work, to better explain these patterns and capture the lived experiences behind them.

## Data Availability

The datasets presented in this article are not readily available because the dataset contains sensitive personal and health-related information and therefore cannot be made publicly available. However, access to the anonymized dataset can be provided for academic and research purposes upon reasonable request. Interested researchers may contact research team to arrange data access in accordance with confidentiality and ethical regulations. Requests to access the datasets should be directed to JK (jan.kizilhan@dhbw.de).
