# Supplementary material for: Exposure to climate-related stressors undermines mental health in the Kurdistan Region of Iraq: a cross-sectional study
Source: Front Public Health. 2026 Jan 12;13:1719584. doi: 10.3389/fpubh.2025.1719584 (PMC12832621; doi:10.3389/fpubh.2025.1719584)
Supplement: Supplementary file 1 [file Supplementary_file_1.docx]

# Survey Questionnaire^[[1]](#footnote-1)^:

# Exposure to Climate-Related Stressors Undermines Mental Health in the Kurdistan Region of Iraq: A Cross-Sectional Study

## Consent^^[[2]](#footnote-2)^^

I confirm that I have read and understood the study information. I am of full age and voluntarily agree to participate in this research.

Yes □ No□

I agree that my data may be used in an anonymized form for future research projects beyond the purpose of this study.

Yes □ No□

I agree to be contacted at my phone number for follow-up inquiries related to the current study or future research projects.

Yes □ No□

## Part 1: Demographic Information

Gender

- Male
- Female

Age _______________

Where do you currently live?

- Erbil
- Duhok
- Sulaymaniyah

Do you live in a rural or urban area?

- Rural area (Land)
- Urban area (City)

Duration of stay in this area in years: _______________

Education of respondent

- No formal education
- Primary
- Secondary
- Higher education

Occupation

- Farmer
- Day labourer
- Small and medium business
- Housewife
- Unemployed
- Academic/Researcher
- Healthcare professional
- Other (please specify): _______________

What sector are you currently employed in?

- Government
- Private sector
- Government and private sector
- None (I am not employed)

Total household monthly income

- Less than 500,000 IQD
- 500,000 – 1,000,000 IQD
- 1,000,001 – 2,000,000 IQD
- More than 2,000,000 IQD

## Part 2: Exposure to Extreme Weather Events and Impact (1)

1.Have you ever faced any extreme weather event during your stay in your region?

Yes □ No□

2. Which type of extreme weather events did you face in your region? Mark all that apply.

- Flood □
- Drought □
- River bank erosion □
- Landslides □
- Earthquake □
- Dust storm □
- Heat wave □
- Wildfire □
- Other □ (please specify): _______________

3.Did you become homeless due to extreme weather events in the past 10 years?

Yes □ No□

4.If yes, frequency of homelessness in past 10 years

Once □ Twice □ □ More than two times

5.Number of homeless days in the past 10 years due to extreme weather events? _______________

## Part 3: Mental Health Measures

### CC-MMDS Scale (2)

Over the past week.. (1= strongly disagree 7= strongly agree)

1. I had to think about the climate change, even without intending to.

① ② ③ ④ ⑤ ⑥ ⑦

1. I have felt anxious, worried, or nervous about the climate change.

① ② ③ ④ ⑤ ⑥ ⑦

1. I have distracted myself to avoid thinking about the climate change.

① ② ③ ④ ⑤ ⑥ ⑦

1. I found it hard to concentrate when I thought about the climate change.

① ② ③ ④ ⑤ ⑥ ⑦

1. I felt depressed at the thought of climate change.

① ② ③ ④ ⑤ ⑥ ⑦

1. I felt helpless when I thought about the climate change.

① ② ③ ④ ⑤ ⑥ ⑦

1. I felt guilty when I thought about the climate change.

① ② ③ ④ ⑤ ⑥ ⑦

1. I felt anger or rage when I thought about the climate change.

① ② ③ ④ ⑤ ⑥ ⑦

1. The extent of the climate change has shaken my worldview.

① ② ③ ④ ⑤ ⑥ ⑦

1. The climate change made me doubt mankind.

① ② ③ ④ ⑤ ⑥ ⑦

1. The climate change made me doubt a just word.

① ② ③ ④ ⑤ ⑥ ⑦

1. I had my doubts about the political approach to climate change.

① ② ③ ④ ⑤ ⑥ ⑦

1. The climate change made me doubt social norms and values.

① ② ③ ④ ⑤ ⑥ ⑦

1. I was afraid of future negative consequences, which could be triggered by the climate change.

① ② ③ ④ ⑤ ⑥ ⑦

1. I felt more uncertainty than usual due to the climate change.

① ② ③ ④ ⑤ ⑥ ⑦

1. The climate change has made it increasingly difficult for me to look positively into the future.

① ② ③ ④ ⑤ ⑥ ⑦

### K10 Scale (3)

In the past 4 weeks..( 1= None of the time, 2= A little of the time, 3= Some of the time, 4= Most of the time, 5= All of the time)

1. In the past 4 weeks, about how often did you feel tired out for no good reason?

① ② ③ ④ ⑤

1. In the past 4 weeks, about how often did you feel nervous?

① ② ③ ④ ⑤

1. In the past 4 weeks, about how often did you feel so nervous that nothing could calm you down?

① ② ③ ④ ⑤

1. In the past 4 weeks, about how often did you feel hopeless?

① ② ③ ④ ⑤

1. In the past 4 weeks, about how often did you feel restless or fidgety?

① ② ③ ④ ⑤

1. In the past 4 weeks, about how often did you feel so restless you could not sit still?

① ② ③ ④ ⑤

1. In the past 4 weeks, about how often did you feel depressed?

① ② ③ ④ ⑤

1. In the past 4 weeks, about how often did you feel that everything was an effort?

① ② ③ ④ ⑤

1. In the past 4 weeks, about how often did you feel so sad that nothing could cheer you up?

① ② ③ ④ ⑤

1. In the past 4 weeks, about how often did you feel worthless?

① ② ③ ④ ⑤

### GAD-7 Scale (4)

Over the last 2 weeks, how often have you been bothered by the following problems? (0= Not at all, 1= Several days, 2= More than half the days, 3= Nearly every day)

1. Feeling nervous, anxious or on edge

⓪ ① ② ③

1. Not being able to stop or control worrying

⓪ ① ② ③

1. Worrying too much about different things

⓪ ① ② ③

1. Trouble relaxing

⓪ ① ② ③

1. Being so restless that it is hard to sit still

⓪ ① ② ③

1. Becoming easily annoyed or irritable

⓪ ① ② ③

1. Feeling afraid as if something awful might happen

⓪ ① ② ③

### PHQ-8 Scale (5)

Over the last 2 weeks, how often have you been bothered by any of the following problems? (0= Not at all, 1= Several days, 2= More than half the days, 3= Nearly every day)

1. Little interest or pleasure in doing things

⓪ ① ② ③

1. Feeling down, depressed, or hopeless

⓪ ① ② ③

1. Trouble falling or staying asleep, or sleeping too much

⓪ ① ② ③

1. Feeling tired or having little energy

⓪ ① ② ③

1. Poor appetite or overeating

⓪ ① ② ③

1. Feeling bad about yourself, or that you are a failure, or have let yourself or your family down

⓪ ① ② ③

1. Trouble concentrating on things, such as reading the newspaper or watching television

⓪ ① ② ③

1. Moving or speaking so slowly that other people could have noticed? Or the opposite – being so fidgety or restless that you have been moving around a lot more than usual

⓪ ① ② ③

### Abbreviated PCL-5 (8-item version) (6)

In the past month, how much were you bothered by: (0=Not at all, 1=A little bit, 2=Moderately, 3=Quite a bit, 4=Extremely)

1. Repeated, disturbing, and unwanted memories of the stressful experience?

⓪ ① ② ③ ④

1. Feeling very upset when something reminded you of the stressful experience?

⓪ ① ② ③ ④

1. Avoiding memories, thoughts, or feelings related to the stressful experience?

⓪ ① ② ③ ④

1. Avoiding external reminders of the stressful experience (for example, people, places, conversations, activities, objects, or situations)?

⓪ ① ② ③ ④

1. Having strong negative beliefs about yourself, other people, or the world (for example, having thoughts such as: I am bad, there is something seriously wrong with me, no one can be trusted, the world is completely dangerous)?

⓪ ① ② ③ ④

1. Loss of interest in activities that you used to enjoy.

⓪ ① ② ③ ④

1. Feeling jumpy or easily startled?

⓪ ① ② ③ ④

1. Having difficulty concentrating?

⓪ ① ② ③ ④

## References

1. Kabir, M. I., Rahman, M. B., Smith, W., Lusha, M. A. F., & Milton, A. H. (2016). Climate change and health in Bangladesh: A baseline cross-sectional survey. *Global Health Action, 9(1)*, 29609. <https://doi.org/10.3402/gha.v9.29609>

2. Beckord, J., Krakowczyk, J. B., Gebhardt, N., Geiser, L. S., Kamler, K., Nikendei, C., Skoda, E.-M., Teufel, M., & Bäuerle, A. (2024). Development and validation of a climate change version of the man-made disaster-related distress scale (CC-MMDS). *The Journal of Climate Change and Health, 20*, 100356. <https://doi.org/10.1016/j.joclim.2024.100356>

3. Kessler, R. C., Andrews, G., Colpe, L. J., Hiripi, E., Mroczek, D. K., Normand, S.-L., Walters, E. E., & Zaslavsky, A. M. (2002). Short screening scales to monitor population prevalences and trends in non-specific psychological distress. Psychological Medicine, 32(6), 959–976. <https://doi.org/10.1017/S0033291702006074>

4. Spitzer, R. L., Kroenke, K., Williams, J. B. W., & Löwe, B. (2006). A brief measure for assessing generalized anxiety disorder: The GAD-7. *Archives of Internal Medicine, 166*(10), 1092–1097. <https://doi.org/10.1001/archinte.166.10.1092>

5. Kroenke, K., Strine, T. W., Spitzer, R. L., Williams, J. B. W., Berry, J. T., & Mokdad, A. H. (2009). The PHQ-8 as a measure of current depression in the general population. *Journal of Affective Disorders, 114*(1–3), 163–173. <https://doi.org/10.1016/j.jad.2008.06.026>

6. Price, M., Szafranski, D. D., van Stolk-Cooke, K., & Gros, D. F. (2016). Investigation of abbreviated 4 and 8 item versions of the PTSD Checklist 5. *Psychiatry Research, 239*, 124–130. <https://doi.org/10.1016/j.psychres.2016.03.014>

1. The questionnaire was formatted and administered in KoBo Toolbox. [↑](#footnote-ref-1)
2. Prior to obtaining informed consent, relevant study information was provided in KoBo Toolbox. [↑](#footnote-ref-2)
